# Immunogenic properties of empty pcDNA3 plasmid against zoonotic cutaneous leishmaniasis in mice

**DOI:** 10.1371/journal.pone.0263993

**Published:** 2022-02-15

**Authors:** Hossein Montakhab-Yeganeh, Reza Shafiei, Mehdi Najm, Leila Masoori, Ashok Aspatwar, Alireza Badirzadeh

**Affiliations:** 1 Molecular Medicine Research Center, Hormozgan Health Institute, Hormozgan University of Medical Sciences, Bandar Abbas, Iran; 2 Department of Biochemistry, Faculty of Medicine, Hormozgan University of Medical Sciences, Bandar Abbas, Iran; 3 Vector-borne Diseases Research Center, North Khorasan University of Medical Sciences, Bojnurd, Iran; 4 Department of Parasitology and Mycology, School of Medicine, Iran University of Medical Sciences, Tehran, Iran; 5 Department of Laboratory Sciences, School of Allied Medical Sciences, Lorestan University of Medical Sciences, Khorramabad, Iran; 6 Faculty of Medicine and Health Technology, Tampere University, Tampere, Finland; National Centre For Cell Science, INDIA

## Abstract

**Background:**

*Leishmania* (L) parasite, the causative agent of zoonotic cutaneous leishmaniasis (ZCL), effectively stimulates the mammalian cells to mount strong humoral responses by enhancing T-helper-2 (Th2)-associated cytokines for its survival. The best strategy to decrease the intensity of infection in the host is induction of cellular immunity.

**Methods:**

We evaluated the effects of the empty bacterial pcDNA3 plasmid on mice infected with *L*. *major* and quantified the immune mediators including IFN-γ, IL-4, IL-10, IgG2a, IgG1, arginase activity and nitric oxide (NO) in the mice. Moreover, the footpad lesion size and parasite load were assessed.

**Results:**

We observed that pcDNA3 could modulate the immune responses in favor of host cells and decrease the disease severity. Th2- associated mediators, including arginase, IL-4, and IL-10 are downregulated, while cellular responses are upregulated in line with an increase in the levels of nitric oxide (NO) and interfero-gamma (IFN-γ). Interestingly, pcDNA3 induced specific Th1-associated antibodies, IgG2a isotype; however, it suppressed the production of humoral IgG1. The stimulation of the immune response by the empty pcDNA3 is able to shift the immune function to predominant cellular responses caused by Th1, and it had a positive effect on the treatment of zoonotic cutaneous leishmaniasis (ZCL).

**Conclusions:**

Altogether, we introduced the pcDNA3 as a potential interfering factor in the modulation of the immune system against ZCL. Since this vector has been widely used as a control group in different studies, we suggest that the potential function of the empty vector should be deeply assessed, as it exerts anti-parasitic effects on mice infected with *L*. *major*.

## Introduction

Leishmaniasis is a neglected tropical infectious disease caused by a vector-borne intracellular protozoan parasite that belongs to the *Leishmania* (L) genus. The disease manifests various clinical features, including cutaneous leishmaniasis (CL), mucocutaneous leishmaniasis (MCL), and visceral leishmaniasis (VL) [1–3]. It is one of the leading endemic protozoan infections in 98 countries, where approximately 350 million people are at risk of infection and cause significant mortality and morbidity [4–6]. CL is the most widespread form of the disease and annually responsible for one million new cases of infection, and about 90% of the cases are reported in tropical and subtropical countries, including Iran, Afghanistan, Algeria, Brazil, Peru, Syria, and Saudi Arabia [7–9].

In recent years, there has been a considerable increase in the knowledge of the interaction among *L*. *major*, its host, and most importantly, the host immune mechanisms. The resistance and susceptibility to *Leishmania* infection are mainly related to the nature of the host immune responses [10]. The attachment of *Leishmania* promastigotes to mononuclear phagocytes, such as macrophages, initiates the infection of mammalian cells, where the promastigotes are transferred into phagolysosomes. In this line, promastigotes are transformed into a flagellate form of parasite or amastigotes (Leishman bodies) and can survive inside the cells. In this condition, amastigotes can unlimitedly proliferate inside the mononuclear phagocytes, promoting the host cells to burst, resulting in the release of *Leishmania* parasites [11, 12].

To date, no effective vaccines are available against the several important protozoan diseases, and existing chemotherapies have various toxic side effects and are very expensive [13, 14]. However, several studies have shown that *Leishmania* can be controlled by vaccination [15, 16]. DNA-based immunotherapy, DNA vaccines, peptide therapy, and gene therapy are some of the approaches to induce protection or survival of the host against not only infectious pathogens but also other disorders, such as cancer and autoimmune diseases [17–19]. It has been demonstrated that cellular or humoral immune responses can be induced by injecting DNA plasmids [20, 21]. These studies suggest that the functional expression of the encoded DNA sequence may result in the modulation of immune responses that might serve as an effective tool for the survival of the host [20, 21].

Biological vectors, especially pcDNA3, have been widely utilized in many studies, such as vaccination for the treatment of various diseases [22, 23]; however, the purpose of these experiments relies on the application of empty pcDNA3 vector as a control strategy and making a comparison with the interventional approaches [22–24]. According to the literature, pcDNA3 is one of the frequently used bacterial expression plasmids mostly used for gene therapy and vaccination due to its versatile properties [25, 26]. Interestingly, DNA vectors, such as bacterial pcDNA3, can induce the production of different cytokines in mammalian cells. This potential capacity has been attributed to immunostimulatory motifs in bacterial plasmids [26, 27]. Studies have shown that these motifs can induce the proliferation and activation of various cytokine genes, such as interferons in mononuclear cells [26].

However, the potential function of the empty bacterial vector in the induction of the host immune responses and consequently, the desired modification in favor of the disease recovery has been neglected. Therefore, we decided to evaluate the potency of the empty bacterial pcDNA3 vector in the prevention of zoonotic cutaneous leishmania (ZCL) caused by *L*. *major*.

## Materials and methods

### Reagents and chemicals

Parasite culture reagents, including RPMI-1640, HEPES, adenosine, hemin, gentamicin, and FBS (fetal bovine serum) were obtained from Sigma-Aldrich (Sigma, Deisenhofen, Germany) and Gibco (Life Technologies GmbH, Karlsruhe, Germany). CYBER Green real-time PCR master mix was purchased from Qiagen (Hilden, Germany). All reagents and solutions were prepared using deionized water by a Milli-Q ultrapure device (Milli-Q System, Millipore, Molsheim, France). All cytokine assay kits were purchased from DuoSet R&D (Minneapolis, USA). Amphotericin B was purchase from CIPLA LTD (Mumbai, India). The EndoFree Plasmid Maxi kit was procured from Invitrogen (Leek, The Netherlands). The BCA protein assay kit was purchased from Thermo Fisher (Waltham, Massachusetts, USA).

### Parasites and pcDNA3

In this study, *L*. *major* (MRHO/IR/75/ER) was employed to infect BALB/c mice. In order to promote *L*. *major* parasites to a virulent and infectious form, parasites were collected from the footpads of infected BALB/c mice. Promastigotes were cultured in the RPMI-1640 medium supplemented with 5% FBS, 40mM HEPES, 0.1mM adenosine, 0.5 μg/ml hemin and 50 μg/ml gentamicin and then incubated at room temperature. The transformed promastigotes were regularly sub-cultured and monitored daily. In order to infect BALB/c mice, a total of 3×10^6^ *L*. *major* promastigotes at the stationary phase (5-day subculture) were isolated and inoculated subcutaneously to the left hind footpad. The bacterial plasmid pcDNA3.1 (+), (pcDNA3), was overexpressed in *Escherichia coli* (*E*. *coli*) and then extracted in a large scale using the EndoFree Plasmid Maxi kit, according to the manufacturer’s protocol [24, 28].

### Ethics approval and consent to participate

All of the experimental procedures, including the maintenance, intervention, and handling were approved by the Institutional Animal Care and Research Advisory Committee of the Iran University of Medical Sciences, Tehran, Iran which in accordance with the international Council for Laboratory Animal Science (ICLAS: https://iclas.org/guidelines-for-researchers/). Furthermore, this study was carried out in compliance with the ARRIVE guidelines (https://arriveguidelines.org).

### Therapeutic schedules and footpad swelling measurement

Female susceptible BALB/c mice (4–6 weeks old; weighing 18–20 g), were obtained from the Pasteur Institute of Iran, Tehran, Iran, and maintained under standard conditions (relative humidity 60%, 24–27°C temperature, 12h:12h light: dark cycles). The animals had free access to normal rodent chow and water.

Mice were infected by the subcutaneous injection of 3×10^6^ promastigotes of *L*. *major* to the left footpad. As displayed in Table 1, animals were divided into four groups (n = 5 per each group), including GI: receiving pcDNA3 as an experimental group (50 μg/mice; via intralesional route; twice weekly), GII: receiving Amphotericin B as a positive control group (8 mg/kg; via intraperitoneal route; daily for two weeks), GIII: being infected with 3×10^6^ promastigotes without receiving ant treatments and coined as a negative control group, and GIV: being infected with 3×10^6^ promastigotes and received phosphate-buffered saline (PBS) (50 μl/mice; via the intralesional rout; twice weekly) as an additional negative control group. Three weeks post-infection, the treatment was initiated and continued for three weeks (the duration of the experiment was six weeks). The rate of footpad swelling (an increase in the thickness and width of ulcer) at the parasite injection site was weekly measured post-infection using a metric calliper and recorded until the end of the experiment.

**Table 1:** *Leishmania major* infected BALB/c mice groups treated by empty bacterial pcDNA3 plasmid.

Groups (G)	Treated with	Concentrations	Route of administration	Treatment duration
GI	pcDNA3	50 μg	Intralesional	Twice weekly for 3 weeks
GII	Amphotericin B	8 mg/kg	Intraperitoneal	Daily for 2 weeks
GIII	No treatment	-	-	-
GIV	PBS 1X	50 μl	Intralesional	Twice weekly for 3 weeks

After 24h of the last session of the measurement of footpad swelling, five mice from each group were selected and anesthetized intraperitoneally using ketamine (20 mg/ml)/xylazine (2.5 mg/ml) and then they were scarified by cervical dislocation. The spleen, foot popliteal lymph nodes draining from the lesion site, and footpad were isolated carefully for cytokine assay, parasite counts, and arginase activity, respectively. All of the experiments were performed twice in separate experiments.

### Nitric oxide (NO) measurement and arginase activity at the site of infection

The spleens of sacrificed mice were removed aseptically for the evaluation of NO using the Griess Reagent Kit, as described previously [29, 30]. In brief, the resected tissue was homogenized in the Dulbecco’s Modified Eagle Medium (DMEM) phenol red-free medium treated using ammonium-chloride-potassium (ACK) lysis buffer (0.15 M NH_4_Cl, 1 mM KHCO_3_, and 0.1 mM Na_2_EDTA) for 5 minutes. Then, the homogenate was washed three times with the DMEM phenol red-free medium and centrifuged at 580 rcf for 7 minutes. A total of 3×10^6^ splenocytes/ml was stimulated in the presence of 10 μg/ml *L*. *major* frozen/thawed (F/T) promastigote antigen (Ag), 5 μg/ml Concanavalin A, as a positive control, and the culture medium, as a negative control followed by incubation at 37°C in a 5% CO_2_-humidified atmosphere. After 5 days, the supernatants were collected for the measurement of nitric oxide.

Infected left footpads of BABL/c mice were homogenized for the analysis of the enzymatic activity of arginase. First, the protein concentrations were quantified using the BCA protein assay kit, according to the manufacturer’s instructions. The assay of arginase activity was performed as described earlier [31]. In brief, after centrifugation, 25 μl of the footpad homogenate was lysed using a lysis buffer and incubated at 56°C for 10 minutes to activate the enzyme. After that, 25 μl of L-arginine, as a substrate for the arginase enzyme, was added to lysates, and the mixture was incubated at 37°C for 60 minutes in order to complete the conversion of the substrate. The reaction was stopped by adding the stopping solution containing H2SO4, H3PO4, and H2O (1:3:7 v/v/v, respectively) into the mixture. In the next step, isonitrosopropiophenone (ISPF) was added to the mixture and incubated at 100°C for 45 minutes. Finally, the concentration of urea was measured spectrophotometrically, at a wavelength of 540 nm, as the product of the arginase enzyme activity. One unit of arginase activity is defined as the amount of enzyme that catalyzes the production of 1 μmol urea per min at 37°C.

### Parasite burden quantification

The lymph nodes of mice were isolated and used for the extraction of genomic DNA using the QIAamp DNA Mini Kit (Qiagen, Hilden, Germany), according to the manufacturer’s instructions. The quality and quantity of the extracted genomic DNA were spectrophotometrically evaluated using a nanodrop instrument (Thermo Scientific, USA). Quantitative real-time PCR (Rotor-Gene Q, Qiagen) was performed by the amplification of two sets of primers targeting a region of kinetoplastid minicircle DNA of *L*. *major* named RV1 (forward: 5′-CTTTTCTGGTCCCGCGGGTAGG-3′), and RV2 (reverse: 5′-CCACCTGGCCTATTTTACACCA-3′) as described in our previous study [10].

### Cytokine assay in splenocytes of infected mice

Five mice from each group were sacrificed for the isolation of spleens. The isolated spleens were homogenized in DMEM and then added to the ACK lysis buffer to eliminate erythrocytes of the splenocyte suspension. Following the stimulation of splenocytes with 10 μg/ml *L*. *major* F/T Ag, as described for the measurement of nitric oxide [10], the level of IFN-γ, IL-4, and IL-10 were assessed in all experimental groups. The cytokine assay was carried out in duplicates using a sandwich ELISA kit (DuoSet, R&D System, USA), according to the manufacturer’s instructions. The final concentration was calculated based on the standard curve drawn for each cytokine [10].

### Anti-leishmanial antibody assessment (IgG2a and IgG1)

The serum levels of IgG_2a_ and IgG_1_ were evaluated using HRP-conjugated antibodies against IgG2a and IgG1. To this aim, Maxi-Sorp plates (Greiner, Germany) were coated with 10 μg/ml *L*. *major* F/T Ag overnight and then blocked with 1% BSA at room temperature for 2 hours. After that, 100 μl (at a dilution of 1:50) of serum was added and incubated at 37°C for about 2 hours. The plates were washed, and anti-mouse IgG1-HRP or IgG2a-HRP (1: 10,000; Southern-Biotech, Canada) was added to each plate. After 2 h incubation at 37°C, the plates were rinsed again, and 20 μl of the TMB micro-well peroxidase substrate solution (KPL, Gaithersburg, MD) was added to each sample. Finally, the plates were incubated for 20 minutes at 37°C, and the reaction was blocked by adding 1% sodium dodecyl sulfate. The absorbance of each group was measured at a wavelength of 405 nm.

### Statistical analysis

All of the obtained values were represented as the mean ± standard deviation (SD) of two separate experiments. Each experiment was carried out at least two times. The statistical analysis was performed using Graphpad Software version 8 and Microsoft Excel 2016. The difference between the experimental groups was analyzed by one-way analysis of variance (ANOVA) and Kruskal-Wallis test depending on whether the data were normally distributed or not, respectively. The student’s T-test and Mann-Whitney test were performed for parametric and non-parametric variables, respectively. The p-value of less than 0.05 was statistically considered significant.

## Results

### Reduction in footpad swelling as a result of pcDNA3 intervention

**Fig 1:**
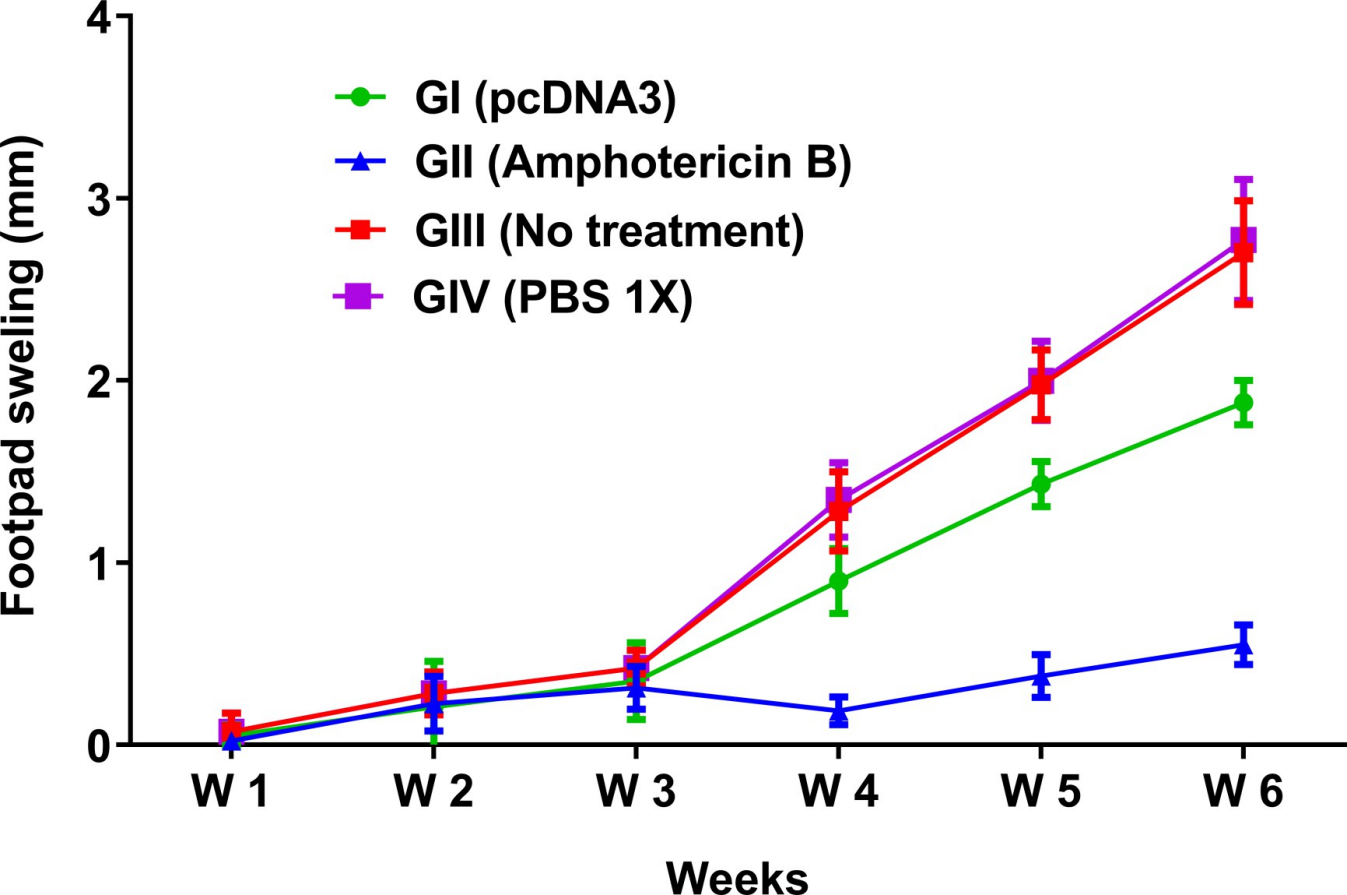
Experimental footpad swelling of infected BALB/c mice in the infection site with *L*. *major* before and post treatment with bacterial plasmid pcDNA3 in comparison with various control groups. The footpad swelling was recorded every week from 1–6 weeks post infection by means of a metric caliper and measuring the increase in the footpad thickness and width. All data have been reported in the current study as the mean ± SD of two independent experiments.

The bacterial plasmid pcDNA3 exhibited no toxicity or mortality in BALB/c mice. Also, other clinical signs, such as the bodyweight reduction, diarrhea, sleepiness, and hair loss, were monitored for one week since the intralesional injection of pcDNA3. As of the first day of infection of mice with *L*. *major*, footpad swelling was weekly monitored and continued up to six weeks. The statistical analysis showed that in the pcDNA3-treated group (GI) the degree of footpad swelling was significantly (p< 0.05) lower than both negative control groups GIII (no treatment) and GIV (PBS), while the rate of footpad swelling was 2-fold higher than the positive control group GII (Amphotericin B) (Fig 1). As shown in Fig 1, a significant (p< 0.05) decrease was found in footpad swelling of the pcDNA3-treated group (GI) compared with the negative control groups GIII (no treatment) and GIV (PBS).

### Overproduction of NO and suppression of arginase activity

**Fig 2:**
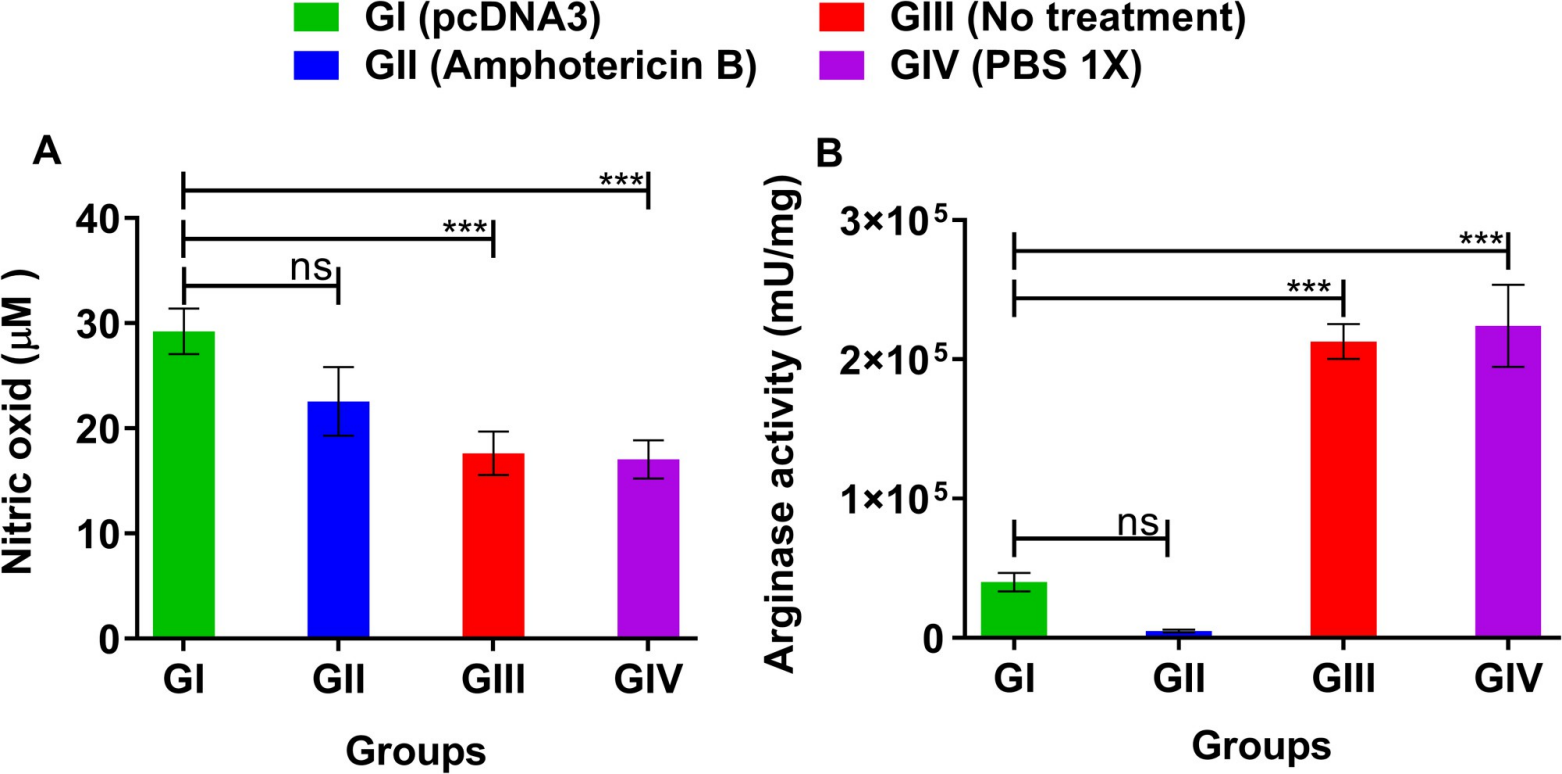
Nitric oxide (NO) production and arginase activity in the susceptible BALB/c mice infected by *L*. *major* three weeks post treatment with bacterial plasmid pcDNA3 in comparison with various control groups in the splenocytes and footpads, respectively. (A) NO (μmol/L) was assessed by using Griess protocol; (B) ARG (mU/mg) level was quantified via microplate method. All data have been reported in the current study as the mean ± SD of two independent experiments; ns: not significant (***p < 0.*001*).

The clearance of the intracellular parasite, *Leishmania*, inside the host cells and importantly, its long-term survival are directly mediated by two main enzymes, namely inducible nitric oxide synthase (iNOS) and arginase, respectively. The amino acid arginine is directly metabolized by these two enzymes expressed inside the host macrophages. A group of cell-mediated Th1 cytokines, such as IFN-γ, trigger the production of nitric oxide by upregulating iNOS, while Th2 cytokines, such as IL-4, increase the arginase activity [10]. Our results demonstrated that the level of nitric oxide is increased in the pcDNA3-treated group (Fig 2A), whereas the activity of arginase is significantly decreased compared with the negative control groups (Fig 2B). Arginase activity showed a very clear inverse association with NO levels in this experiment (Fig 2). This observation could be attributed to the activation of iNOS and subsequent NO production, mediated by a competitive enzyme, arginase, that consumes arginine, as a substrate.

### Parasite burden in the pcDNA3-treated group

**Fig 3:**
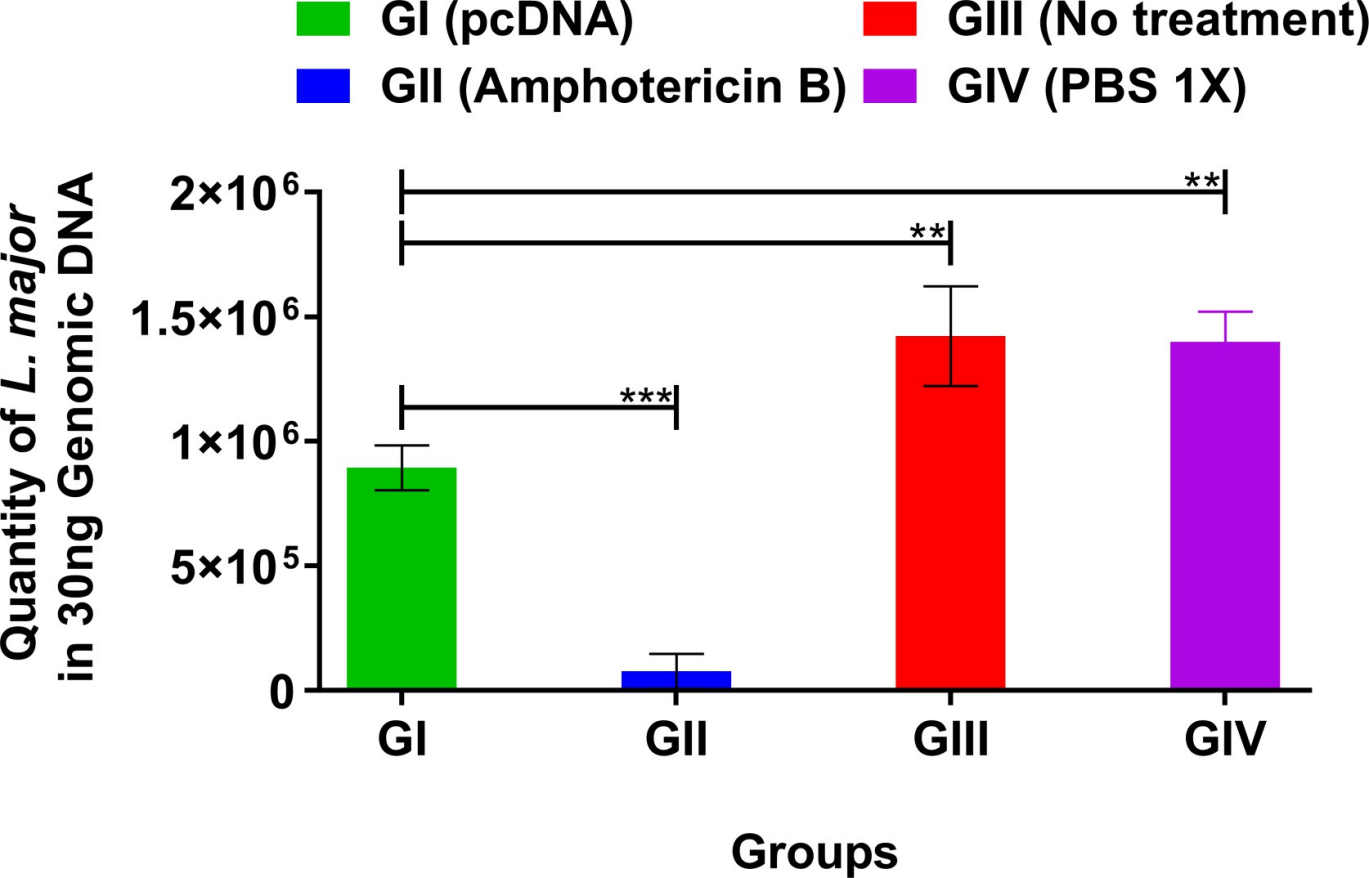
Quantification of *L*. *major* parasite burden in the isolated lymph nodes of susceptible BALB/c mice three weeks post treatment with bacterial plasmid pcDNA3 3.1(+) (pcDNA3) in comparison with various control groups by using quantitative real-time PCR (qPCR). qPCR targeting the conserved region of *Leishmania* kinetoplast DNA (kDNA) performed for exact determination of parasite number at the lymph nodes of mice (number of parasites per 30 ng of DNA). All data have been reported in the current study as the mean ± SD of two independent experiments (**p < 0.*01*, ***p < 0.*001*).

An absolute load of *L*. *major* parasites in homogenized lymph nodes of all experimental groups was quantified at three weeks following the intervention with pcDNA3 (Fig 3). The statistical analysis indicated a significant (p< 0.005) decrease in parasite load in the pcDNA3-treated group (GI) in comparison with the negative control groups (GIII) (GIV). As shown in Fig 3, the highest level of parasite load was detected in both negative control groups (GIII) (GIV), while the lowest number of parasites was observed in the Amphotericin B-treated group as a positive control group (GII).

### Cytokine assay in splenocytes of infected mice

**Fig 4:**
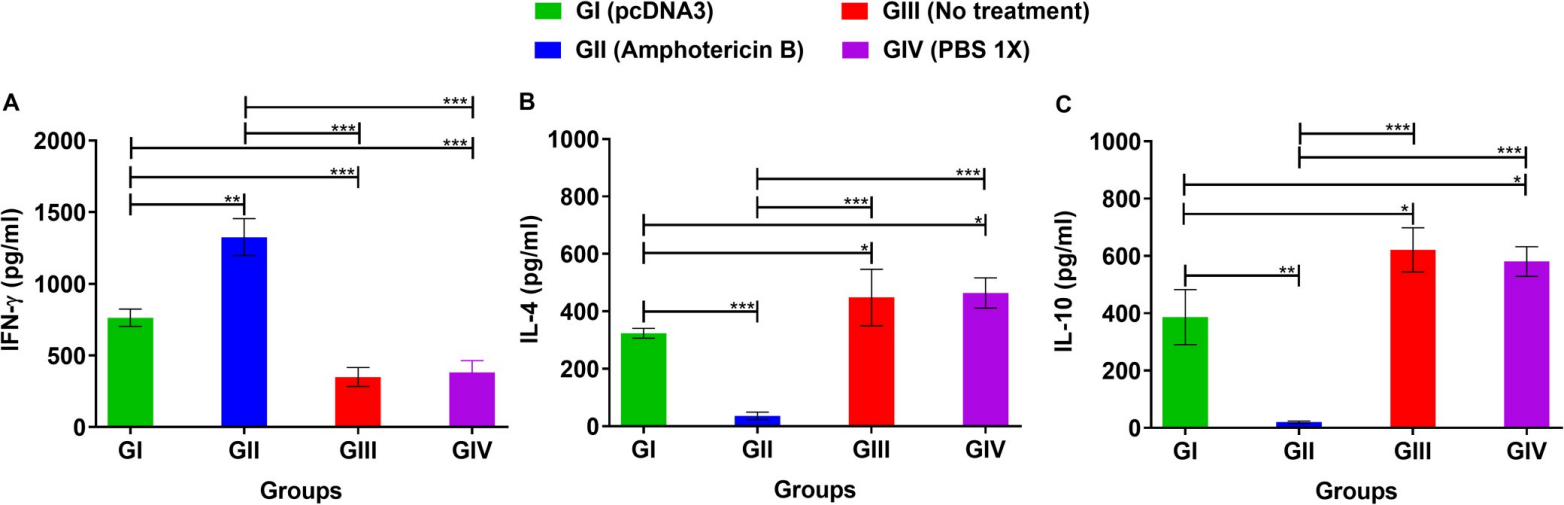
Cytokines production profile of stimulated splenocytes in infected BALB/c mice with *L*. *major* three weeks post treatment by bacterial plasmid pcDNA3 in comparison with various control groups. After homogenizing the spleen, 3×10^6^ splenocytes/ml were stimulated with 10 μg/ml *L*. *major* frozen/thawed antigen and the three main immune system cytokines (IFN- γ, IL-4 and IL-10) determined using ELISA system. (A) IFN-γ (Th1 cytokine), (B) IL-4 and (C) IL-10 (Th2 cytokines) were quantified. All data have been reported in the current study as the mean ± SD of two independent experiments (*p < 0.*05*, **p < 0.*01*, ***p < 0.*001*).

**Fig 5:**
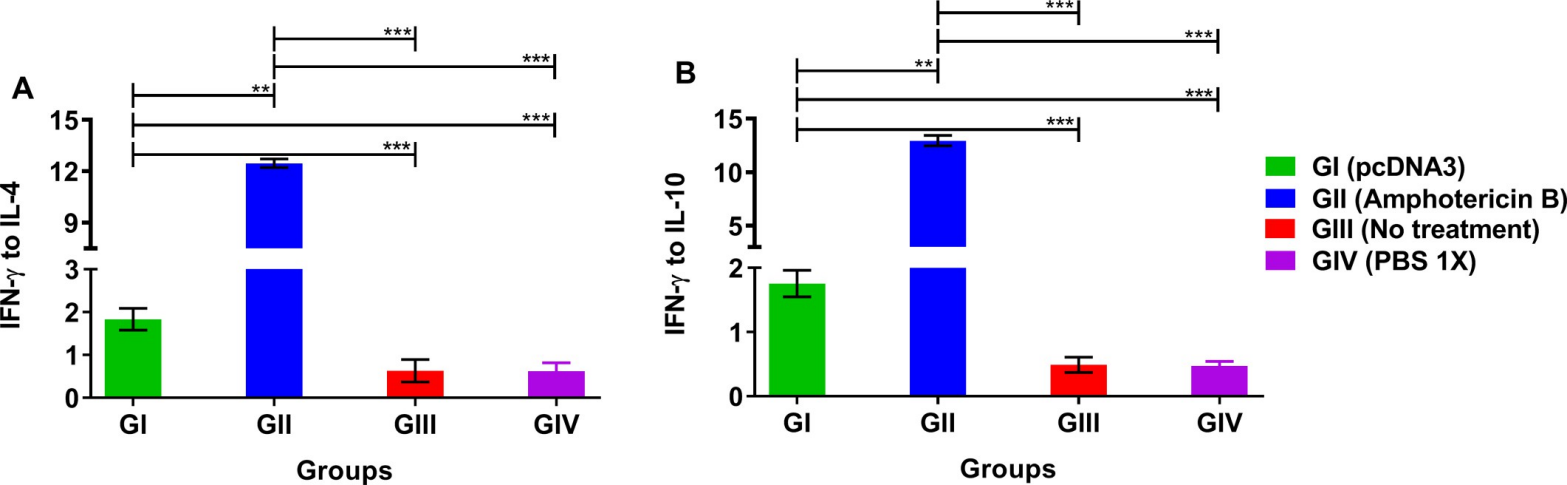
Different cytokines ratios in the supernatant of frozen/thawed antigen stimulated in the *L*. *major* infected BALB/c mice splenocytes three weeks post treatment by bacterial plasmid pcDNA3 in comparison with various control groups. (A) Ratio of IFN- γ to IL-4 and (B) IFN- γ to IL-10. All data have been reported in the current study as the mean ± SD of two independent experiments (**p < 0.*01*, ***p < 0.*001*).

In order to characterize the specific types of the induced immune system responses (Th1 or Th2), the levels of IFN-γ, as one of the main Th1 cytokines, as well as IL- 4 and IL-10, as major Th2 cytokines, were quantified in the supernatant of F/T Ag-stimulated splenocytes (Figs 4 and 5). Our data indicated that pcDNA3 (GI) and Amphotericin B (GII) significantly induced the expression of IFN-γ (Fig 4A), while they decreased the expression of IL-4 and IL-10 (Fig 4B and 4C) compared with the negative control groups. Furthermore, the stimulation of splenocytes with pcDNA3 (GI) and Amphotericin B (GII) caused a significant increase in the ratios of IFN- γ/IL-4 and IFN- γ/IL-10, implying the shift of immune response toward the production of Th1 cytokines (Fig 5).

### Anti-leishmanial antibody assessment (IgG2a and IgG1)

**Fig 6:**
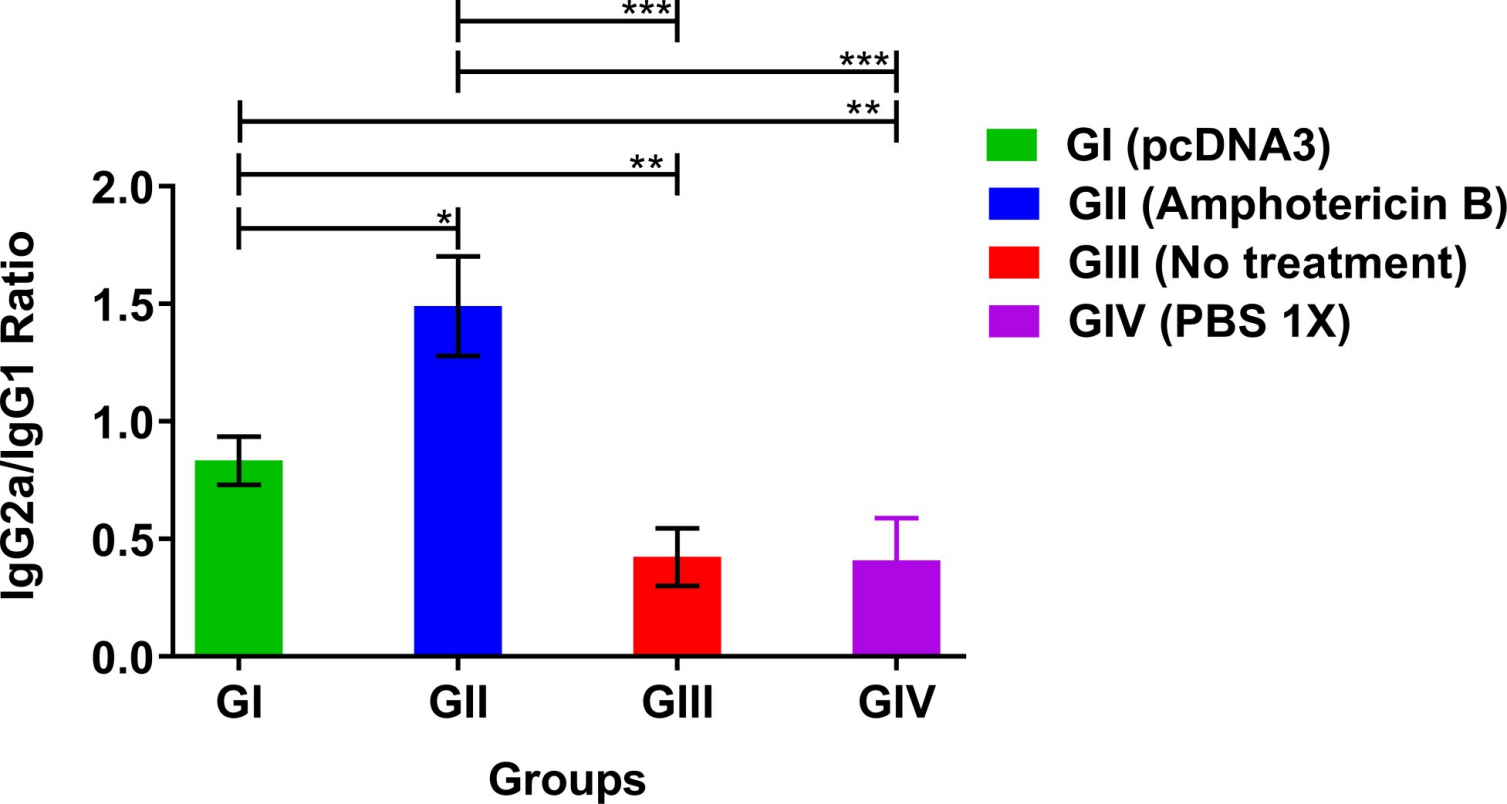
Evaluation of IgG2a/IgG1 ratios using anti IgG2a and IgG1 HRP conjugated anti-bodies in *L*. *major* infected BALB/c mice three weeks post treatment by bacterial plasmid pcDNA3 in comparison with various control groups. All data have been reported in the current study as the mean ± SD of two independent experiments (*p < 0.*05*, **p < 0.*01*, ***p < 0.*001*).

Studies have demonstrated that IFN-γ (Th1-inducing cytokines), along with IL-4, and IL-10 (Th2-inducing cytokines) induce isotype switching from IgG to either IgG2a or IgG1, respectively [32, 33]. IgG2a/IgG1 are considered significant biomarkers for Th1 and Th2 responses against protozoan parasites such as *Leishmania* [32]. Therefore, to confirm this hypothesis, IgG1, and IgG2a antibody responses were determined against F/T Ag, and their ratios were calculated (Fig 6). The ratio of IgG2a/ IgG1 in pcDNA3-treated (GI) and Amphotericin B-treated (GII) groups were increased by 2 folds and 3 folds compared with the negative control groups, respectively, denoting the shift of the immune response towards the production of Th1 cytokines. The higher ratio of IgG2a/IgG1 was found in mice treated with pcDNA3 against *L*. *major* infection and showed high protective capacity in the immune response of these animals.

## Discussion

Despite significant advances in the treatment of different types of leishmaniasis, available tools must overcome various barriers, including *Leishmania* parasites, sand-fly vectors, and, most importantly, the complexity of the host to discover effective drugs. Furthermore, despite the fact that much effort has been made to find appropriate prophylactic agents or vaccines against CL, there is no effective vaccine for the mammalian host, especially humans [34]. To date, chemotherapy is the only therapeutic approaches used for the management of *Leishmania*, and therefore, extensive research is required to discover qualified anti-leishmanial drugs. Although numerous compounds, such as natural and synthetic nanoparticles, have been used for the treatment of *Leishmania* [35], the toxicity of these drugs as well as the resistance of the parasite has limited the efficacy of these therapeutic agents [36, 37].

Many studies have used recombinant organisms that are able to express different cytokines when co-expressed with the pcDNA3 plasmid, such as CXCL-10 (pcDNA3-CXCL-10-EGFP), which has been used as an immunotherapeutic agent for the treatment of mice infected with *Leishmania* [24, 38]. However, no study examined the empty pcDNA3 plasmid as an immunostimulatory agent for the treatment of ZCL in BALB/c mice. Of note, in different studies, pcDNA3 has been used as a control group without considering the potential function of this empty vector as an immunogenic compound. Therefore, we decided to examine the effects of the empty pcDNA3 vector, for the first time, on the attenuation of ZCL in BALB/c mice. Interestingly, our results demonstrated that the immune cell response was shifted from the production of Th2 cells towards Th1 cells in BALB/c mice infected with pathogenic *L*. *major* and treated with pcDNA3. The study confirms the finding of other experiments in which the Th1-associated response showed a good prognosis during *Leishmania* infection, while Th2 responses were correlated with poor prognosis and disease severity [39].

Our findings showed that the empty pcDNA3 plasmid, devoid of any inserted sequences, decreased the severity of lesions caused by leishmanial infection compared with the negative control groups. In addition, in mice treated with the empty pcDNA3, the lesion size and parasite load were significantly decreased in comparison with negative control groups. The empty pcDNA3 plasmid is capable of inducing specific and direct effects on the murine immune system. Correspondingly, in animals treated with the empty pcDNA3 plasmid, the concentration of nitric oxide was increased, while the arginase activity was decreased compared with negative control groups. The production of IFN-γ was significantly elevated in the pcDNA3-treated group, whereas the levels of IL-4 and IL-10 were significantly lowered compared with negative control groups. Finally, the higher ratio of IgG2a/IgG1 was found in mice infected with *L*. *major* and then treated with pcDNA3, leading to increased protective capacity in the animal immune response.

According to our results, the empty pcDNA3 plasmid reduced *Leishmania* parasite in infected BALB/c mice as the parasite load was significantly decreased at the site of infection, as well as in the foot-draining popliteal lymph nodes draining from the lesion site. These results demonstrated that pcDNA3 has therapeutic potential against *L*. *major* amastigotes via the modulation of the immune response, without causing harmful side effects on the host cells [40, 41]. In contrast to our findings, several studies showed that the lesion size and parasite load in animals treated with the empty pcDNA3 vector did not significantly cause any difference [40, 41].

It is now known that nitric oxide, IFN-γ, and IgG2a are Th1-associated mediators, protecting mammalian cells from leishmanial infection, while, arginase, IL-4, IL-10, and IgG1 are Th2-associated mediators, which are produced in favor of the proliferation and virulence of the parasite [42–45]. Our experiments showed that when the animals were infected with *L*. *major* and then treated with the empty pcDNA3 vector, the cytokine balance was shifted towards a Th1 response, suggesting that the therapeutic effects of empty pcDNA3 vector might be related to the activation of Th1 cells. The expression analysis of IFN-γ in stimulated splenocytes showed that it tends to be increased compared with that of the uninfected control group. We found out that the difference between infected and uninfected BALB/c mice was statistically significant when the IFN-γ production was induced in-vivo by the empty pcDNA3. A study performed by Johansson et al. showed that pcDNA3 could induce IFN-α in swine whole blood cells in-vitro [26, 28]. We showed that the arginase activity was decreased, whereas the production of nitric oxide was increased in the pcDNA3-treated group which was beneficial for the host immune cells in the form of helping macrophages to effectively eradicate *Leishmania* parasites. This response may be due to the properties of the vector that is discussed in the following paragraph. Contrarily, one study has shown that the animals immunized with empty pcDNA3, as a control group, exhibited a marked increase in humoral immune cytokines, such as IL-4 and IL-10 [41]. In that study, a comparison was made between the efficacy of the recombinant cysteine proteinase or rLdccys1 (experimental group) with empty pcDNA3 (control group). They showed that the use of rLdccys1 led to a higher number of Th1 cells compared with those treated with pcDNA3. Obviously, our data were in contrast to their findings.

The precise mechanism of action on how the empty bacterial pcDNA3 vector boosted the host immune responses is not fully understood. This effect might be mediated via a motif, namely CpG, inside the bacterial vector [27]. The effect of the pcDNA3 vector as bacterial DNA on the mammalian host immune system is of interest. Bacterial pcDNA3 contains immunostimulatory motifs consisting of a central unmethylated CpG dinucleotide (5’-C-phosphate-G-3’), which is flanked by two 5’-purines and two 3’-pyrimidines [24, 27]. Although the CpG motifs frequently exist in different bacterial genomes, it seems their frequency is lower in vertebrate genomes because of the presence of a balance between the rate of methylation and unmethylation of the CpG motif [23, 44]. According to the literature, the CpG motif stimulates the cellular immune responses as a result of Th1-associated mediators in mammalian cells [24, 46, 47].

Additionally, several experiments have shown that bacterial DNA modulates autoimmune reactions [24, 48]. Boccacio et al. have demonstrated that non-coding plasmid DNA inhibits experimental allergic encephalomyelitis via the activation of IFN-γ [27]. CpG motifs have been used as an adjuvant to enhance the cellular responses in various disorders, such as cancer, and it has a significant immunostimulatory effect on the induction of cell death in cancer cells via activating cytotoxic T cells [49]. Bacterial CpG motif binds to toll-like receptor 9 (TLR-9), which is expressed by different immune system cells, such as macrophages, dendritic cells and natural killer (NK) cells. This motif is known to activate lymphocytes and induce them to secrete a verity of cytokines, including IL-6, IL-12, and IFN-γ [49]. Quintana et al. have reported that CpG motif is able to induce the expression of IFN-γ [24]. Another study has reported that the empty pcDNA3 vector that contains a CpG motif can stimulate Th1 responses in-vivo; therefore, it is capable of reducing the development of metabolic diseases such as diabetes [24]. For this reason, other researchers must take precise attention to this fact that the empty bacterial pcDNA3 vector can induce the host immune system to induce Th1 responses; thus, we suggest further studies must be performed to elucidate the effects of the empty bacterial pcDNA3 vector.

It has been that CpG motifs possess a unique structure that stimulates antigen‐presenting cells (APCs) to produce distinct proinflammatory cytokines such as IL-12 via the stimulation of TLR-9 [50]. CpG motifs contain several repeats of unmethylated C and G nucleotides [51], mimicking *Leishmania*-associated molecular patterns and are recognized by TLR9. This phenomenon activates a number of signaling cascades such as c-Jun N-terminal kinase (JNK) and NF-kB. TLR9 is required for the functionality of CpG to induce NF-kB activation, resulting in the stimulation of the activation of the IL-12 p40 promoter. Thus, CpG motifs play a significant role in the activation of Th1 response in BALB/c mice infected with *L*. *major* [52]. Studies have shown that TLRs can be implicated in distinct pathogen recognition and trigger sufficient immune effector functions. TLR9 belongs to the pathogen recognition receptors, interacting with intracellular parasites such as *Leishmania* [50], and it is crucial for the induction of NK cells through the innate immune response in cutaneous leishmaniasis [53, 54]. Furthermore, distinct studies have shown that vaccination of BALB/c mice by major *Leishmania* antigen namely soluble leishmanial antigen (SLA)+CpG-ODN-stimulated several DCs (SLA–CpG–DCs) had an strong protection against *L*. *donovani* visceral infection [50, 55].

Along with CpG motifs which have been used as an adjuvant to enhance the cellular responses, other compounds were applied for developing a successful therapeutic adjuvant against *Leishmania* infection. One of these therapeutic adjuvant was combination of *Mycobacterium indicus pranii* with heat induced *Leishmania* promastigotes (HIP) [56]. By using this combinational adjuvant Th1 response was enhanced by Th17 activation and IL-6 induction as one of the key cytokines and suppressed the T regulatory cells immunosuppressive activities in therapeutic strategy against drug-resistant *L*. *donovani* infection [56]. In another experiment, immunogenicity of a *Leishmania* virulence factor including the *Leishmania* eukaryotic Elongation Factor-1 beta (EF1b) protein as DNA vaccine or recombinant protein-adjuvant, had scrutinized parasitologically and immunologically as a vaccine candidate against L. infantum infection in-vitro and in-vivo. Using EF1b alone or protein-adjuvant developed Th1 response and enhanced parasitological protection [57]. In a study, a TLR agonist (gardiquimod), as an adjuvant was applied for vaccination against VL due to *L*. *donovani* infection. Immunized BALB/c had the least *Leishmania* parasite burden, high delayed-type hypersensitivity responses, and significantly boosted Th1 cytokines levels (IFN-γ, IL-12, and IL-17), nitric oxide and IgG2a [58].

In the current study, Amphotericin B, as a positive control, significantly induced the production of Th1 cells compared with pcDNA3. Although Amphotericin B has strong anti-leishmanial activity and does not need to participate in the host immune response, our finding demonstrated that it stimulated Th1-associated mediators through the production of IFN-γ and NO as well as the suppression of arginase activity and the expression of IL-4 and IL-10. Studies have shown that Amphotericin B exerts anti-leishmanial activity in T cell-deficient and nude mice [59, 60]. Besides, various experiments indicated that Amphotericin B has anti-fungicidal properties via the induction of specific cytokines, production of inflammatory mediators, and activation of different cells, but its anti-leishmanial activity is independent of the immune system and directly affects the survival of parasites [60]. It has been indicated that the combination of Amphotericin B with several immunomodulators, such as IL-12 and Human Neutrophil Peptide 1 (HNP1), exhibits synergistic effects on the induction of cell death in leishmanial infection. One possible explanation for its mechanism of action is that the combinatory use of these agents remarkably enhances the Th1 responses and increases the expression of HNP1. It seems that Amphotericin B, in addition to the anti-leishmanial properties, is able to stimulate Th1-associated cell response through increasing the expression of HNP1, leading to increased efficacy of therapeutic agents [61]. Further studies are needed to understand the precise mechanisms underlying the role of Amphotericin B in the induction of Th1-associated cell response. It has been shown that IL-12 enhances the responsiveness of Amphotericin B in mice knockout for the IFN-γ gene; hence, the synergistic effect of IL-12 and Amphotericin B in mice is dependent on IFN-γ. Thus, IL-12 regulates host IFN-γ–dependent and–independent responses and enhance the anti-leishmanial activity of Amphotericin B [62].

## Conclusions

The administration of the empty pcDNA3 vector to mice susceptible to leishmaniasis stimulated the immune responses and effectively induced Th1-associated cell responses during the treatment of leishmaniasis. This study provides a novel potency of pcDNA3 as an immunostimulatory agent and confirms the ability of this vector to induce protective immunity against *L*. *major* infection in BALB/c mice. The current study suggests that the design of different DNA vaccines using pcDNA3 could be a potential choice for the treatment of *L*. *major*. Our results demonstrate that the empty pcDNA3 vector interferes with the outcomes of other therapeutic options used for the treatment of *L*. *major* and exhibits synergistic effect when used in combination with other compounds. Thus, the use of the empty pcDNA3 vector, as a control group, may not be suitable in studies conducted on the immunostimulatory effects of different drugs.
